# Greater need but reduced access: a population study of planned and elective surgery rates in adult mental health service users

**DOI:** 10.1017/S2045796024000131

**Published:** 2024-03-18

**Authors:** G. Sara, J. Hamer, P. Gould, J. Curtis, P. Ramanuj, T. A. O’Brien, P. Burgess

**Affiliations:** 1 InforMH, System Information and Analytics Branch, NSW Ministry of Health, Sydney, NSW, Australia; 2 Northern Clinical School, Sydney Medical School, University of Sydney, Sydney, NSW, Australia; 3 School of Psychiatry, University of NSW, Sydney, NSW, Australia; 4 Mid North Coast Local Health District, Coffs Harbour, NSW, Australia; 5 London Spinal Cord Injury Centre, Royal National Orthopaedic Hospital, London, UK; 6 RAND Europe, London, UK; 7 Cancer Institute NSW, Sydney, NSW, Australia; 8 Medicine & Science, University of New South Wales, Sydney, NSW, Australia; 9 School of Public Health, University of Queensland, Brisbane, NSW, Australia

**Keywords:** chronic conditions, cross-sectional study, epidemiology, health service research

## Abstract

**Aims:**

Timely access to surgery is an essential part of healthcare. People living with mental health (MH) conditions may have higher rates of chronic illness requiring surgical care but also face barriers to care. There is limited evidence about whether unequal surgical access contributes to health inequalities in this group.

**Methods:**

We examined 1.22 million surgical procedures in public and private hospitals in New South Wales (NSW), Australia, in 2019. In a cross-sectional study of 76,320 MH service users aged 18 and over, surgical procedure rates per 1,000 population were compared to rates for 6.23 million other NSW residents after direct standardisation for age, sex and socio-economic disadvantage. Rates were calculated for planned and emergency surgery, for major specialty groups, for the top 10 procedure blocks in each specialty group and for 13 access-sensitive procedures. Subgroup analyses were conducted for hospital and insurance type and for people with severe or persistent MH conditions.

**Results:**

MH service users had higher rates of surgical procedures (adjusted incidence rate ratio [aIRR]: 1.53, 95% CI: 1.51–1.56), due to slightly higher planned procedure rates (aIRR: 1.22, 95% CI: 1.19–1.24) and substantially higher emergency procedure rates (aIRR: 3.60, 95% CI: 3.51–3.70). Emergency procedure rates were increased in all block groups with sufficient numbers for standardisation. MH service users had very high rates (aIRR > 4.5) of emergency cardiovascular, skin and plastics and respiratory procedures, higher rates of planned coronary artery bypass grafting, coronary angiography and cholecystectomy but lower rates of planned ophthalmic surgery, cataract repair, shoulder reconstruction, knee replacement and some plastic surgery procedures.

**Conclusions:**

Higher rates of surgery in MH service users may reflect a higher prevalence of conditions requiring surgical care, including cardiac, metabolic, alcohol-related or smoking-related conditions. The striking increase in emergency surgery rates suggests that this need may not be being met, particularly for chronic and disabling conditions which are often treated by planned surgery in private hospital settings in the Australian health system. A higher proportion of emergency surgery may have serious personal and health system consequences.

## Introduction

Surgery is an essential part of healthcare. Limited or delayed surgical access can lead to mortality and morbidity from untreated illness, more advanced illness at the time of surgery, worse surgical outcomes and higher healthcare costs (Bergmark *et al.*, 2022; Fu *et al.*, 2020). Therefore, surgical access variation has been used as a measure of healthcare disparity within and between countries (Meara *et al.*, 2015; Rose *et al.*, 2015; Taylor *et al.*, 2022). Access variation may lead to ‘surgical deserts’ even in well-funded health systems (Uribe-Leitz *et al.*, 2018), and vulnerable groups may face particular financial or systemic barriers to accessing surgical care (Royal Australasian College of Surgeons, 2017).

People living with mental health (MH) conditions have reduced life expectancy and increased rates of chronic health conditions, partly due to barriers to accessing healthcare (Firth *et al.*, 2019). MH conditions have been associated with lower rates of specific surgical procedures including cardiac re-vascularisation, joint replacement, transplantation, bowel cancer surgery and breast-conserving cancer surgery (Baillargeon *et al.*, 2011; Bhattacharya *et al.*, 2022; Chang *et al.*, 2020; Copeland *et al.*, 2015; Kisely *et al.*, 2009, 2007; Li *et al.*, 2011). A handful of studies have examined a broader range of surgical procedures in MH cohorts, finding higher rates for some procedures. In South London, people receiving MH care were 40–80% more likely to have emergency cardiac (Brooks *et al.*, 2022) or vascular surgery (Ghani *et al.*, 2021). US veterans with MH diagnoses had higher rates of emergency procedures such as partial amputations (Copeland *et al.*, 2015). In 10 European countries, people reporting depressive symptoms in a population survey also reported higher rates of chronic health conditions and an approximately 50% higher rate of surgical care (Peytremann-Bridevaux *et al.*, 2008).

To integrate these findings, we hypothesise that people with MH conditions have more need for surgery, due to higher rates of chronic health conditions, but they also face barriers to access. This is reflected in lower rates of planned and evidence-based surgery for conditions such as heart disease or cancer but increased rates of some emergency surgery. To test this hypothesis, we examine all surgical procedures for the population of New South Wales (NSW), Australia, comparing rates for people with and without prior specialist MH care after adjusting for differences in age, gender and socio-economic disadvantage. We examine overall surgery rates and compare planned and emergency surgery rates for different specialty (procedure block) groups and specific access-sensitive procedures. We focus on surgical procedures during hospitalisation for acute and chronic non-communicable diseases where surgery is commonly part of treatment. We include subgroup analysis of MH service users to examine possible relationships between severity of mental illness and rates of surgical access.

## Methods

### Service setting and context

The study examined all public and private hospital admissions for adults in NSW, Australia (adult population 6.3 million in 2019). Australians have free access to government-funded (‘public’) hospitals, which provide approximately 42% of all surgery and more than 85% of emergency surgery (Australian Institute of Health and Welfare, 2020). Most planned or elective surgery (66% in 2018–19) occurs in private hospitals (Australian Institute of Health and Welfare, 2020). State governments provide waiting lists for elective surgery in public hospitals. Slightly under half of Australian adults purchase optional private health insurance (Australian Prudential Regulation Authority, 2023), enabling faster access to surgery via private admission to private or public hospitals.

MH services in Australia are also mainly funded by national and state governments. We defined MH service users as people receiving care from (i) public or private hospital MH inpatient units or (ii) public community or outpatient MH services. Public (state government operated) community MH services provide acute and emergency community MH care as well as long-term community MH care for people with severe or enduring illness. The study did not have data from office-based MH services by private practitioners.

### Data linkage

Data from NSW public and private hospitals, public community MH services and Register of Births Deaths and Marriages were linked by the NSW Centre for Health Record Linkage. Probabilistic record linkage used individuals’ names, birth-dates, addresses and health service identifiers. The linkage is designed to give a false positive linkage rate of around 5 per 1,000 records. More details on datasets and linkage methods are provided elsewhere (Sara *et al.*, 2019).

### Data sources and selection criteria

We examined NSW public and private hospital episodes ending between 1 January and 31 December 2019, avoiding COVID-19 related disruptions to planned surgery in NSW in 2020 and 2021 (Sutherland *et al.*, 2020). First, we identified in-scope same-day or overnight hospitalisations of adult NSW residents. We excluded episodes with primary diagnoses of mental or behavioural conditions; accident or injury (where procedures may reflect treatment of self-harm attempts); conditions not typically treated by surgery (infectious diseases, blood and immune disorders, pregnancy and puerperium) and episodes with missing diagnoses. Episodes missing address or sex were also excluded because they could not be included in rate standardisation.

We extracted all surgical procedures from in-scope hospital episodes. NSW hospital diagnoses and procedures are coded by Health Information Managers based on clinical records and discharge summaries. Diagnoses are recorded using the International Classification of Diseases, version 10, Australian Modification (Australian Consortium for Classification Development, 2017), and procedure codes recorded using the Australian Classification of Health Interventions (ACHI) (Independent Hospital and Aged Care Pricing Authority, 2019). Procedure codes are organised in a three-level hierarchical structure, with more than 39,000 procedure codes grouped into approximately 1,000 ‘blocks’ and 15 higher level ‘block groups’. For example, the procedure *38306-00: Percutaneous insertion of transluminal stent into single coronary artery* sits within block *671: Transluminal coronary angioplasty with stenting*, in the *Cardiovascular Procedures* block group.

The ACHI also includes non-surgical interventions. To exclude these, two authors (GS and JH) reviewed all block names to exclude codes which were primarily diagnostic (endoscopy, radiology, lumbar puncture, skin biopsy, etc.), staffing-related (allied health consultations, etc.) or non-surgical (dialysis, urinary catheterisation, external respiratory support, pain management, anaesthetics, etc.). We also excluded procedure block groups for electroconvulsive therapy, obstetrics, radiology, radiation oncology, cancer, dental and other/unspecified procedures. After data extraction, the same authors reviewed (i) procedure blocks accounting for 95% of procedures within each diagnostic group, procedure block group and hospital type (public or private) and (ii) the top 500 individual procedure codes. Following review, additional exclusions were applied for procedures whose full text description indicated a non-surgical or diagnostic purpose (including terms such as ‘administration’, ‘biopsy’, ‘examination’, ‘diagnostic’, ‘anaesthetic’ or ending in ‘… scopy’).

### Primary outcome: surgical procedure rates

The rate of surgical procedures expressed as procedures per 1,000 population per year was calculated overall and separately for planned and emergency admissions defined using an ‘Emergency Status’ variable applying to the hospital episode. If an admission included multiple surgical procedures, all were included in rate calculations.

Rates were calculated for block groups, the 10 most frequent procedure blocks within each block group, and selected individual procedures used as measures of differential surgical access in Australian national reporting: appendicectomy, cataract extraction, cholecystectomy, coronary angioplasty, coronary artery bypass graft, haemorrhoidectomy, hip replacement, hysterectomy, inguinal herniorrhaphy, knee replacement, prostatectomy, septoplasty and tonsillectomy (Australian Commission on Safety and Quality in Health Care and Australian Institute of Health and Welfare, 2017; Australian Institute of Health and Welfare, 2020).

### MH service user status

MH service users were defined as NSW residents aged 18–100 years who had any MH hospitalisation or public community MH contact during the study period (Jan–Dec 2019) or the preceding 2 years (Jan 2017 to Dec 2018). MH hospitalisations included public or private hospital admissions with a primary diagnosis of a non-organic MH condition (ICD-10 codes F10–F99) or at least 1 day in a designated MH unit. Community contacts included face-to-face or telehealth contacts with NSW public community MH services. Non-NSW residents, administrative contacts, case conferences and contacts by community teams with hospital inpatients were excluded.

For subgroup analysis, ‘severe or persistent mental illness (SPMI)’ was defined as either (i) any diagnosis of schizophrenia, schizoaffective disorder, bipolar disorder or psychotic depression or (ii) more than 2 years of continuous MH service contact.

### Independent variables

Sex and area of residence were defined at the first contact in the observation period. To align with reference population data and for consistency between procedure and MH service datasets, ages were calculated at 1 July 2019. Socio-economic disadvantage was estimated from the person’s area of residence using the Australian Bureau of Statistics Index of Relative Socio-economic Disadvantage (Australian Bureau of Statistics, 2006). This index scores Australian geographical areas using 17 census-derived variables measuring income, welfare support, education, home ownership, employment, household structure and English language proficiency. Scores were divided at the quintiles to create five groups of roughly equal population size.

### Statistical analysis

Data assembly and analysis were conducted in SAS Enterprise Guide v7.15. Surgical procedure rates per 1,000 person-years were calculated separately for MH and non-MH groups. Procedure rates for each block group, procedure block and access-sensitive procedure were first calculated separately for each stratum of age (18–24, 25–34, 35–44, 45–54, 55–64, 65–75, 75–84, 85+), sex and quintile of socio-economic disadvantage. For rate calculations, the denominator for the MH service user group was the total count of MH service users. The population (non-MH) denominator was the Australian Bureau of Statistics reference population for 2019 after subtracting the number of MH service users for each sex, age group and disadvantage quintile. Adjusted incidence rate ratios (aIRRs) and 95% log-normal confidence intervals were calculated by direct standardisation using the SAS procedure ‘Proc STDRATE’. To avoid unreliable estimates, standardised rates were not calculated for procedure blocks or codes with 20 or fewer events in the MH cohort.

## Results

**Figure 1.**
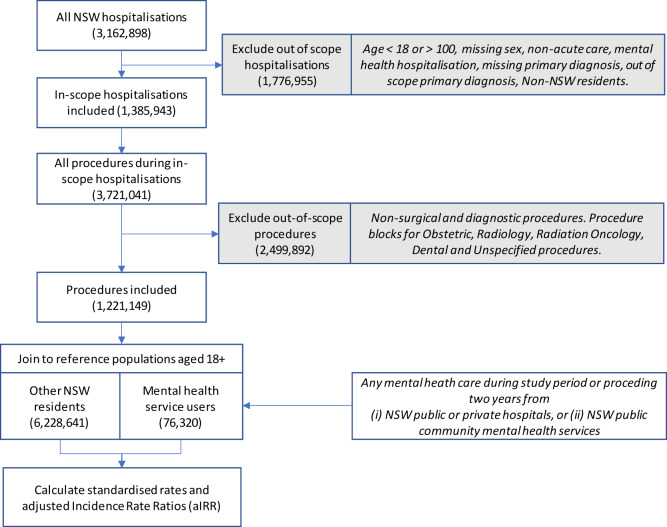
Study flowchart.

We examined 3.16 million hospitalisations in NSW public and private hospitals in 2019. After excluding out-of-scope hospitalisations and procedures, the analysis included 1.22 million procedures (see study flowchart in Fig. 1). We identified 76,320 MH service users aged 18 and above. In the overall NSW population, surgical procedures increased steadily with age (Table 1) and varied for different strata of disadvantage. Compared to the NSW population, MH service users were younger and more likely to live in more disadvantaged regions.

**Table 1.** Population, number of surgical procedures and unadjusted procedure rates per 1,000 population in mental health service users compared to other NSW residents, by stratum of sex, age group and socio-economic disadvantage. NSW public and private hospitals, January–December 2019

	Mental health service users			Other NSW residents		
	People, *n* (%)	Procedures, *n* (%)	Rate (95% CI)	People, *n* (%)	Procedures, *n* (%)	Rate (95% CI)
Total	76,320 (100%)	19,304 (100%)	253 (249−257)	6,228,641 (100%)	1,201,845 (100%)	193 (193−193)
Sex						
Female	38,841 (51%)	9,620 (50%)	248 (243−253)	3,167,594 (51%)	623,765 (52%)	197 (196−197)
Male	37,479 (49%)	9,684 (50%)	258 (253−264)	3,061,047 (49%)	578,080 (48%)	189 (188−189)
Age group						
18−24	12,138 (16%)	1,030 (5%)	85 (80−90)	736,428 (12%)	33,669 (3%)	46 (45−46)
25−29	7,952 (10%)	915 (5%)	115 (108−123)	605,590 (10%)	29,121 (2%)	48 (48−49)
30−34	7,806 (10%)	913 (5%)	117 (109−125)	594,799 (10%)	38,492 (3%)	65 (64−65)
35−39	7,879 (10%)	1,256 (7%)	159 (151−168)	560,463 (9%)	48,939 (4%)	87 (87−88)
40−44	7,152 (9%)	1,241 (6%)	174 (164−183)	499,063 (8%)	55,027 (5%)	110 (109−111)
45−49	7,195 (9%)	1,694 (9%)	235 (224−247)	519,523 (8%)	71,318 (6%)	137 (136−138)
50−54	5,987 (8%)	1,733 (9%)	289 (276−303)	471,889 (8%)	77,929 (6%)	165 (164−166)
55−59	5,065 (7%)	1,820 (9%)	359 (343−376)	489,314 (8%)	97,136 (8%)	199 (197−200)
60−64	3,994 (5%)	1,723 (9%)	431 (411−452)	443,290 (7%)	115,963 (10%)	262 (260−263)
65−69	3,092 (4%)	1,826 (9%)	591 (563−618)	390,079 (6%)	140,435 (12%)	360 (358−362)
70−74	2,793 (4%)	1,970 (10%)	705 (674−736)	339,048 (5%)	164,559 (14%)	485 (483−488)
75−79	2,050 (3%)	1,379 (7%)	673 (637−708)	238,742 (4%)	141,099 (12%)	591 (588−594)
80−84	1,511 (2%)	960 (5%)	635 (595−676)	166,827 (3%)	102,863 (9%)	617 (613−620)
85+	1,706 (2%)	844 (4%)	495 (461−528)	173,586 (3%)	85,295 (7%)	491 (488−495)
Disadvantage quintile						
1 (most)	13,158 (17%)	3,375 (17%)	256 (248−265)	1,126,741 (18%)	178,067 (15%)	158 (157−159)
2	21,032 (28%)	4,966 (26%)	236 (230−243)	1,075,712 (17%)	312,239 (26%)	290 (289−291)
3	14,644 (19%)	3,707 (19%)	253 (245−261)	1,324,373 (21%)	225,971 (19%)	171 (170−171)
4	13,556 (18%)	3,587 (19%)	265 (256−273)	1,270,761 (20%)	215,436 (18%)	170 (169−170)
5 (least)	13,930 (18%)	3,669 (19%)	263 (255−272)	1,431,054 (23%)	270,132 (22%)	189 (188−189)

**Figure 2.**
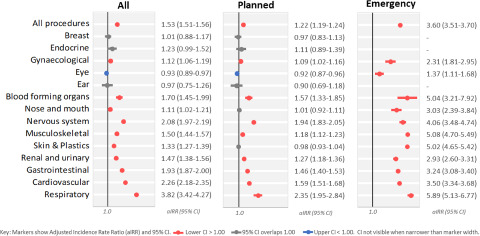
Adjusted incidence rate ratios (aIRRs) for surgical procedures in NSW mental health service users compared to other NSW residents, after standardisation for age sex and socio-economic disadvantage. Rates calculated for all procedures and separately for planned and emergency procedures.

MH service users experienced 19,304 in-scope surgical procedures, a crude rate of 253 per 1,000 population, while other NSW residents (No MH) experienced 1,201,845 procedures (193 per 1,000) (Table 1). In MH service users, surgical procedure rates were slightly higher in males than in females, but the reverse was true in other NSW residents. After standardisation by age, sex and disadvantage (Table 2), the MH group experienced 296 procedures per 1,000 person years compared to 193 per 1,000 in the No MH group (aIRR: 1.53, 95% CI: 1.51–1.56). Relative rates differed by procedure block group (Fig. 2): MH rates were slightly lower for eye procedures (aIRR: 0.93, 95% CI: 0.89–0.97), equivalent to No MH rates for breast, endocrine and ear procedures and higher for all other procedure block groups. The highest relative rates in the MH group were for gastrointestinal, cardiovascular, nervous system and respiratory block groups, with aIRRs from 1.93 to 3.82.

**Table 2.** Number and rate of surgical procedures by procedure block group for NSW mental health service users compared to other NSW residents, showing adjusted rate per 1,000 after standardising for age, sex and socio-economic disadvantage

	All		Planned		Emergency	
**Number of procedures**	**MH**	**No MH**	**MH**	**No MH**	**MH**	**No MH**
**All procedures**	**19,304**	**1,201,845**	**13,207**	**1,041,574**	**6,097**	**160,271**
Breast	198	17,267	186	16,985	12	282
Endocrine	92	7,345	83	7,193	9	152
Gynaecological	1,123	80,611	1,051	78,234	72	2,377
Eye	1,964	229,530	1,867	222,596	97	6,934
Ear	66	6,348	59	6,101	7	247
Blood forming organs^a^	169	10,178	148	9,787	21	391
Nose and mouth	598	44,315	518	42,127	80	2,188
Nervous system	1,529	67,200	1,350	62,864	179	4,336
Musculoskeletal	2,572	159,293	1,849	145,913	723	13,380
Skin and plastics	2,189	151,609	1,411	138,620	778	12,989
Renal and urinary	1,149	78,019	856	68,492	293	9,527
Gastrointestinal	4,015	188,582	2,236	138,392	1,779	50,190
Cardiovascular	3,286	152,448	1,473	98,939	1,813	53,509
Respiratory	354	9,100	120	5,331	234	3,769
**Rate per 1,000 (95% CI)**	**MH**	**No MH**	**MH**	**No MH**	**MH**	**No MH**
**All procedures**	**295.5 (291.1−299.8)**	**192.7 (192.3−193.0)**	**202.9 (199.3−206.5)**	**167.0 (166.6−167.3)**	**92.6 (90.1−95.0)**	**25.7 (25.6−25.8)**
Breast^b^	2.8 (2.4−3.2)	2.8 (2.7−2.8)	2.6 (2.2−3.0)	2.7 (2.7−2.8)	—	—
Endocrine^b^	1.4 (1.1−1.7)	1.2 (1.2−1.2)	1.3 (1.0−1.6)	1.2 (1.1−1.2)	—	—
Gynaecological	14.5 (13.7−15.4)	13.0 (12.9−13.0)	13.7 (12.8−14.5)	12.6 (12.5−12.7)	0.9 (0.7−1.1)	0.4 (0.4−0.4)
Eye	34.2 (32.6−35.7)	36.7 (36.6−36.9)	32.7 (31.1−34.2)	35.6 (35.5−35.8)	1.5 (1.2−1.8)	1.1 (1.1−1.1)
Ear^b^	1.0 (0.7−1.2)	1.0 (1.0−1.0)	0.9 (0.6−1.1)	1.0 (1.0−1.0)	—	—
Blood forming organs^a^	2.8 (2.3−3.2)	1.6 (1.6−1.7)	2.5 (2.0−2.9)	1.6 (1.5−1.6)	0.3 (0.2−0.5)	0.1 (0.1−0.1)
Nose and mouth	7.9 (7.2−8.6)	7.1 (7.1−7.2)	6.8 (6.2−7.4)	6.8 (6.7−6.8)	1.1 (0.8−1.3)	0.4 (0.3−0.4)
Nervous system	22.4 (21.2−23.6)	10.8 (10.7−10.9)	19.6 (18.5−20.7)	10.1 (10.0−10.2)	2.8 (2.4−3.3)	0.7 (0.7−0.7)
Musculoskeletal	38.4 (36.9−40.0)	25.5 (25.4−25.7)	27.5 (26.2−28.8)	23.4 (23.3−23.5)	10.9 (10.1−11.7)	2.1 (2.1−2.2)
Skin and plastics	32.3 (30.8−33.7)	24.3 (24.2−24.4)	21.8 (20.6−23.0)	22.2 (22.1−22.3)	10.5 (9.7−11.3)	2.1 (2.1−2.1)
Renal and urinary	18.4 (17.3−19.5)	12.5 (12.4−12.6)	13.9 (12.9−14.9)	11.0 (10.9−11.1)	4.5 (3.9−5.0)	1.5 (1.5−1.6)
Gastrointestinal	58.6 (56.6−60.5)	30.3 (30.1−30.4)	32.5 (31.0−33.9)	22.2 (22.1−22.3)	26.1 (24.8−27.4)	8.1 (8.0−8.1)
Cardiovascular	55.2 (53.3−57.2)	24.4 (24.3−24.5)	25.2 (23.9−26.5)	15.8 (15.7−15.9)	30.0 (28.6−31.5)	8.6 (8.5−8.6)
Respiratory	5.6 (5.0−6.2)	1.5 (1.4−1.5)	2.0 (1.6−2.4)	0.9 (0.8−0.9)	3.6 (3.1−4.0)	0.6 (0.6−0.6)

a Blood forming organs group includes procedures on bone marrow, lymphatic tissues or spleen.

b Standardised rates were not calculated when there were fewer than 20 procedures in the mental health service user group.

Emergency procedures comprised 14% of total in-scope procedures. The MH group had slightly higher planned procedure rates (aIRR: 1.22, 95% CI: 1.19–1.24) but substantially higher emergency procedure rates (aIRR: 3.60, 95% CI: 3.51–3.70) (Fig. 2). Using the counts in Table 2, we calculated the proportion of total procedures in each block which occurred during emergency admissions. This ranged from 2% for breast and endocrine procedure block groups to 42% for respiratory procedures. The relative rate of emergency procedures was increased in all block groups with sufficient numbers for standardisation. aIRRs ranged from 1.37 (95% CI: 1.11–1.68) for emergency eye procedures to 5.89 (95% CI: 5.13–6.77) for emergency respiratory procedures.

We examined the 10 most frequent procedure blocks within each block group (Supplementary Tables S2a–d). Emergency procedure incidence rates were higher in the MH group in all blocks with sufficient numbers for standardisation. Adjusted rate ratios for emergency procedures exceeded 4.0 for 12 procedure blocks, particularly for cardiovascular (myocardial preservation and coronary artery bypass grafting), skin and plastics (excision, debridement, drainage and skin grafting) and respiratory groups (tracheostomy, endoscopic biopsy and foreign body removal). For planned procedures, MH service users had lower standardised rates for 10 procedure blocks, particularly in musculoskeletal (shoulder reconstruction, arthroscopic meniscectomy, arthroscopic excision of the knee and arthroplasty of the knee), skin and plastics (excision of skin lesions, local skin flap procedures and eyelid excision) and eye (extraction of crystalline lens and insertion of intraocular lens prostheses) block groups.

Examining individual access-sensitive procedures (Table 3), the MH group had lower rates of planned surgery for cataract repair (aIRR: 0.91, 95% CI: 0.84–0.98) and knee replacement (aIRR: 0.84, 95% CI: 0.71–0.98). In contrast, the MH group had higher rates of planned and emergency surgery for coronary artery bypass grafting, coronary angiography and cholecystectomy. The aIRR for emergency surgery was higher than for planned surgery for all individual procedures with sufficient numbers for standardisation.

**Table 3.** Selected access-sensitive procedures for NSW mental health service users compared to other NSW residents. Number of procedures and adjusted incidence rate ratios (aIRRs) after standardising for age, sex and socio-economic disadvantage

	All procedures			Planned			Emergency		
Procedure	Total	Emerg %	aIRR (95% CI)	No MH (*n*)	MH (*n*)	aIRR (95% CI)	No MH (*n*)	MH (*n*)	aIRR (95% CI)
**Cardiac**									
Coronary artery graft	7,240	35%	3.23 (2.80−3.73)	4,561	99	2.46 (2.00−3.02)	2,456	106	4.67 (3.81−5.71)
Coronary angioplasty	18,533	45%	2.06 (1.84−2.30)	9,927	136	1.51 (1.27−1.79)	8,209	205	2.72 (2.36−3.14)
**Orthopaedic**									
Knee replacement	20,774	1%	0.85 (0.72−0.99)	20,441	157	0.84 (0.71−0.98)	118	3	–
Hip replacement	12,467	2%	1.05 (0.88−1.26)	12,038	111	0.98 (0.81−1.19)	263	12	–
**Gastrointestinal**									
Haemorrhoidectomy	21,733	2%	1.00 (0.88−1.14)	21,014	252	0.98 (0.86−1.12)	411	9	–
Hernia repair	15,657	8%	0.97 (0.82−1.15)	14,174	123	0.84 (0.69−1.01)	1,278	34	2.43 (1.68−3.51)
Cholecystectomy	18,622	27%	1.60 (1.43−1.79)	13,288	203	1.33 (1.15−1.54)	4,931	146	2.35 (1.97−2.80)
Appendicectomy	9,303	441%	1.63 (1.52−1.75)	5,440	85	1.54 (1.22−1.93)	40,190	800	1.67 (1.55−1.80)
**Genito-urinary**									
Hysterectomy	9,400	1%	1.10 (0.91−1.33)	9,159	109	1.06 (0.87−1.29)	111	5	–
Prostatectomy	11,812	2%	1.07 (0.87−1.30)	11,435	96	1.04 (0.85−1.27)	239	5	–
**Eye and ENT**									
Cataract	86,663	1%	0.92 (0.85−0.99)	84,987	693	0.91 (0.84−0.98)	907	12	–
Septoplasty	7,549	1%	1.02 (0.82−1.26)	7,344	94	1.01 (0.82−1.26)	85	2	–
Tonsillectomy	4,752	2%	1.35 (1.09−1.66)	4,515	91	1.29 (1.03−1.60)	110	6	–

In subgroup analysis by hospital and insurance type (Table 4), 62% of in-scope procedures occurred in private hospitals and 63% were covered by private health insurance. The number of privately insured procedures can exceed those in private hospitals because some people receive care as privately insured patients in public hospitals. After adjusting for age, sex and socio-economic disadvantage, MH service users had lower rates of planned procedures in private hospitals (aIRR: 0.97, 95% CI: 0.95–0.99) or funded by private insurance (aIRR: 0.93, 95% CI: 0.90–0.95).

**Table 4.** Surgical procedure rates in mental health service users, subgroup analysis by type of hospital and insurance type. Rates standardised for age, sex and socio-economic disadvantage. It is possible to elect to be treated as a privately insured patient in a public hospital. ‘Other’ insurance status includes workers compensation, motor vehicle accident compensation and military personnel or veterans

		Procedures		Standardised rate		Rate ratio	
		No MH	MH	No MH	MH	aIRR (95% CI)	*p*
**All procedures**							
Hospital type	Public	450,869	11,674	72.3	175.4	2.43 (2.38−2.47)	<0.0001
	Private	750,976	7,630	120.3	120.1	1.00 (0.97−1.02)	0.8497
Insurance status	None	385,716	10,108	61.9	149.5	2.42 (2.37−2.47)	<0.0001
	Private	756,506	7,836	121.2	126.2	1.04 (1.02−1.07)	0.0006
	Other	59,623	1,360	9.6	22.6	2.37 (2.24−2.51)	<0.0001
**Planned procedures**							
Hospital type	Public	309,564	5,932	49.7	89.0	1.79 (1.74−1.84)	<0.0001
	Private	732,010	7,275	117.3	113.9	0.97 (0.95−0.99)	0.0173
Insurance status	None	284,134	5,444	45.6	80.8	1.77 (1.72−1.82)	<0.0001
	Private	703,613	6,534	112.7	104.4	0.93 (0.90−0.95)	<0.0001
	Other	53,827	1,229	8.6	20.9	2.42 (2.28−2.57)	<0.0001
**Emergency procedures**							
Hospital type	Public	141,305	5,742	22.7	86.4	3.81 (3.71−3.92)	<0.0001
	Private	18,966	355	3.0	12.2	4.02 (3.61−4.48)	<0.0001
Insurance status	None	101,582	4,664	16.3	68.6	4.21 (4.08−4.34)	<0.0001
	Private	52,893	1,302	8.5	25.3	2.98 (2.82−3.16)	<0.0001
	Other	5,796	131	0.9	7.1	7.66 (6.39−9.18)	<0.0001

*Note*: Rate = procedures per 1,000 population. aIRR = adjusted incidence rate ratio.

In subgroup analysis (Supplementary Tables S3 and S4), surgical procedure rates differed for people with SPMI compared to other MH service users. Planned surgery rates were lower in people with SPMI (157.6 per 1,000, 95% CI: 152.6–162.6) and higher in MH service users (247.1, 95% CI: 241.6–252.7) compared to other NSW residents (167.0, 95% CI: 166.6–167.3). Relative to other NSW residents, people with SPMI had lower planned surgery rates for eye, musculoskeletal system, nose and mouth procedures. Emergency surgery rates were higher than other NSW residents for people with SPMI (aIRR: 2.19, 95% CI: 2.08–2.30) and other MH service users (aIRR: 4.90, 95% CI: 4.74–5.05). Unexpectedly, other MH service users had a standardised emergency procedure rate (125.9 per 1,000, 95% CI: 121.9–129.8) more than twice that of people with SPMI (56.2 per 1,000, 95% CI: 53.3–59.1). The higher rate of emergency surgery affected most procedure types: people with SPMI had higher rates of emergency surgery than the NSW population for all block groups other than eye surgery (aIRR: 1.19, 95% CI: 0.86–1.64), and other MH service users had higher rates for all procedure blocks with sufficient numbers for standardisation.

A higher proportion of MH service users (9.6%) had at least one procedure, compared to other NSW residents (7.7%) (Supplementary Table S1). Among people with at least one procedure, MH service users had slightly more procedures per person (2.6, other NSW residents 2.5) but the same number of procedures per hospital admission (1.9). Of the MH population, 3.5% experienced at least one emergency surgical procedure, compared to 1.3% of other NSW residents. Among people having any procedure, MH service users also had a higher rate of emergency procedures per person (MH 2.3, other NSW residents 2.0).

## Discussion

It has been argued that ‘access to surgical care and access to healthcare are synonymous’ (Alkire *et al.*, 2015); however, there is no consensus framework for measuring inequalities in surgical access (de Jager *et al.*, 2019; Taylor *et al.*, 2022). We studied over 1.2 million surgical procedures from all public and private hospitals in NSW in 2019, allowing reasonably precise estimates of relative rates for common procedure types. We found that MH service users had substantially higher rates of surgery, primarily due to a 3.6-fold higher rate of emergency surgery. Both within broad groups of procedures and for individual access-sensitive procedures, MH service users had substantially elevated rates of emergency procedures for all procedure types with sufficient data for standardisation, with two- to sixfold higher emergency surgery rates for most procedure groups. Age, sex or socio-economic differences did not explain these elevated rates. Higher rates were mainly due to more MH service users experiencing at least one procedure, rather than a subgroup of MH service users having very high rates.

Higher surgical procedure rates could be explained by greater need, with a higher prevalence of conditions requiring surgical care. We found substantially elevated rates of gastrointestinal, cardiovascular and respiratory procedures, which may reflect a higher prevalence of cardiac, metabolic, alcohol-related or smoking-related conditions in people living with mental ill health (Firth *et al.*, 2019). The findings are consistent with studies showing higher rates of cardiovascular procedures in people with serious mental illness (Brooks *et al.*, 2022; Ghani *et al.*, 2021).

However, the greater relative increase in emergency surgery (aIRR: 3.60) than planned surgery (aIRR: 1.22) suggests that this increased need is not being effectively met. For MH service users, nearly one third (32%) of all procedures occurred during emergency admissions, compared to only one in eight (13%) in other NSW residents. For example, we found that MH service users had similar or slightly lower rates of planned hernia repair than other NSW residents (aIRR: 0.84, 95% CI: 0.69–1.01) but were more than twice as likely to have hernia repair during an emergency admission (aIRR: 2.43). Emergency admissions may be more likely when inaccessible or delayed care leads to advanced or unstable disease, and the ratio of planned to emergency procedures has been proposed as a measure of health system access (Prin *et al.*, 2018). Delayed access to planned care and higher rates of emergency surgery may have serious personal and health system consequences (The Lancet Rheumatology, 2021), and emergency procedures are associated with higher complication rates, worse outcomes and higher mortality (Bergmark *et al.*, 2022; Prin *et al.*, 2018; Vogel *et al.*, 2010).

We found that MH service users had slightly higher rates for many planned surgical procedures, including planned admission for all cardiac procedures (aIRR: 1.59), including coronary artery bypass grafting (aIRR: 2.46), coronary angiography (aIRR: 1.53) and coronary angioplasty (aIRR 1.51). This may appear inconsistent with studies from Canada (Kisely *et al.*, 2009, 2007) and Hong Kong (Chang *et al.*, 2020) reporting lower rates of cardiac procedures in MH cohorts. However, those studies compared procedure rates in people already admitted for coronary heart disease. MH service users may have increased surgical procedure rates at the population level but also have reduced rates relative to clinical need because they have a greater prevalence of conditions requiring urgent care.

By contrast, we found that MH service users had lower rates of planned admission for some procedure types, including ophthalmic surgery, cataract repair, shoulder reconstruction, knee replacement and some plastic surgery procedures. In the Australian health system, many of these typically occur as planned procedures in private hospitals. We found that MH service users had lower rates of surgery in private hospitals or using private insurance, even after adjusting for socio-economic disadvantage. Financial barriers have a major impact on surgical access (Taylor *et al.*, 2022) and may contribute to low rates of joint replacement surgery in US Medicaid recipients with MH conditions (Li *et al.*, 2011). In Australia’s primarily government-funded health system, emergency care is reasonably accessible regardless of ability to pay. However, access to surgery for disabling but non-life-threatening conditions requires gatekeeping and prioritisation by general practitioners and surgeons and may involve costly insurance premiums, substantial out-of-pocket expenses or lengthy waiting lists for free public hospital care. Because of high out-of-pocket costs, Australians living with chronic conditions are more likely to forego healthcare for financial reasons compared to countries with similar health systems (Callander *et al.*, 2017). People living with MH conditions are particularly likely to miss care due to costs (Callander *et al.*, 2017), and there are concerns that Australians with MH conditions may be denied cover or face discriminatory exclusions when seeking private health insurance (Public Interest Advocacy Centre, 2021).

Our findings suggest that for people living with MH conditions, surgery rates are high relative to the broader population but low relative to need. Addressing these gaps requires evidence at several levels. First, surgical need may be reduced by more effective prevention (Bergmark *et al.*, 2022), addressing risk factors such as smoking, inactivity, diet and medication-related weight gain (Firth *et al.*, 2019), or earlier primary care to prevent progression of avoidable chronic conditions (Kim *et al.*, 2019). Second, health systems should understand barriers to ‘indication detection’ (de Jager *et al.*, 2019) or recognition of the need for surgery (Bergmark *et al.*, 2022). These may be reduced by better primary care access or greater awareness of the risks of diagnostic overshadowing of physical symptoms by MH conditions (Jones *et al.*, 2008). Third, once needs are identified, there are many possible clinical, structural and funding barriers to progression to surgery (de Jager *et al.*, 2019; Royal Australasian College of Surgeons, 2017). A systematic review has identified nearly 70 measures of need or access for specific surgical conditions or procedures (de Jager *et al.*, 2019): many could be used to measure health disparities in people living with MH conditions.

In subgroup analysis, we found that people with SPMIs, including people with psychosis, had lower rates of planned surgery but higher rates of emergency surgery than other NSW residents. People with SPMI have particularly high prevalence of chronic conditions (Firth *et al.*, 2019), but despite having increased need are also likely to face additional financial and systemic barriers to accessing planned surgery. The higher emergency surgery rate in other MH service users suggests that people with SPMI face additional barriers to accessing emergency surgery when needed. It also suggests that strategies to improve access to surgical care should consider people with a wide range of MH conditions, not only people living with psychoses and related disorders.

### Limitations

We acknowledge several limitations of this study. First, we defined MH service users via contact with a subset of Australian MH services (public and private hospital care, state-operated community care) which are focused on people living with more acute or severe illness. The results may not apply to the larger group receiving MH care only in primary care or private office-based settings.

Second, we do not have data on procedures in private outpatient or non-hospital day-procedure centres. Care in those settings almost always requires private insurance or out of pocket costs, and so we may be underestimating the gap in access to planned surgery for MH service users.

Third, we attempted to exclude diagnostic and non-surgical procedures; however, the interventions classification used (ACHI) does not include a standard grouping or variable which distinguishes treatment and diagnostic procedures. Different inclusion or exclusion criteria may change our findings for specific procedure blocks or groups.

Fourth, we distinguished planned from emergency surgery using the overall ‘emergency status’ of the admission in which surgery occurred. We did not have access to surgical wait-list data which could also be used to distinguish planned from emergency surgery or quantify time spent awaiting surgery. It is possible for emergency surgery to occur during a planned admission, for example a person may require surgery for a fracture or acute appendicitis during a planned admission for another condition. Therefore, we may have slightly underestimated emergency surgery rates. However, this is infrequent and unlikely to produce substantial error or bias.

Finally, we did not have data on some potentially important co-variates including cultural background and the type or adequacy of prior outpatient medical care. Our measure of socio-economic disadvantage was based on the person’s region of residence rather than on individual income.

## Conclusions

Access to timely surgical care is a critical part of an effective health system. Studies of surgical access in people living with MH conditions have mainly focused on specific surgical indications or procedures. Our study of population-wide surgical procedure rates shows that people living with MH conditions have higher surgical procedure rates, mainly due to much higher rates of emergency surgical care. These findings suggest that for people living with MH conditions, surgery rates are high relative to the broader population but low relative to need. Even in a country with universal healthcare, barriers to timely surgery may have substantial individual and health system impacts.

## Supporting information

Sara et al. supplementary material Sara et al. supplementary material

## Data Availability

No data are available. Access to NSW Health data is available to researchers only with the specific approval of the NSW Population and Health Services Research Ethics Committee (www.cancer.nsw.gov.au/research-and-data/nsw-population-health-services-research-ethics-com). That approval does not permit sharing of unit record data with other researchers.

## Supplementary material

The supplementary material for this article can be found at https://doi.org/10.1017/S2045796024000131.
