# Supplementary material for: Greater need but reduced access: a population study of planned and elective surgery rates in adult mental health service users
Source: Epidemiol Psychiatr Sci. 2024 Mar 18;33:e12. doi: 10.1017/S2045796024000131 (PMC10951789; doi:10.1017/S2045796024000131)
Supplement: Sara et al. supplementary material [file S2045796024000131sup001.docx]

#

# Greater need but reduced access: a population study of planned and elective surgery rates in adult mental health service users.

# SUPPLEMENTARY MATERIAL

1. Numbers of people, hospital admissions and procedures by mental health group and planned/emergency status.
2. Procedure rates, top 10 blocks within each block group:
   1. Cardiovascular, Gastrointestinal and Respiratory
   2. Muskuloskeletal, Skin and Plastics, Genito-urinary
   3. Nervous system, Eye, Nose and Mouth
   4. Gynaecological, Breast, Endocrine, Blood-forming organs
3. Subgroup analysis, surgical procedure rates and counts in people with Severe and Persistent Mental Illness, other mental health service users and the rest of the NSW adult population.
4. Subgroup analysis: adjusted Incidence Rate Ratios for people with Severe or Persistent Mental Illness (SPMI) and other mental health service users compared separately to other NSW adults without MH service use.

S1: Numbers of people, hospital admissions and procedures by mental health group and planned/emergency status

| Procedure type | **All** | | | **Emergency** | | | **Planned** | | |
| --- | --- | --- | --- | --- | --- | --- | --- | --- | --- |
| Group | **All** | **No MH** | **MH** | **All** | **No MH** | **MH** | **All** | **No MH** | **MH** |
| Population | 6,304,963 | 6,228,643 | 76,320 | 6,304,961 | 6,228,641 | 76,320 | 6,304,963 | 6,228,643 | 76,320 |
| People with any procedure | 485,656 | 478,322 | 7,334 | 83,307 | 80,646 | 2,661 | 423,121 | 417,882 | 5,239 |
| Percent with any procedure | 7.7% | 7.7% | 9.6% | 1.3% | 1.3% | 3.5% | 6.7% | 6.7% | 6.9% |
| Hospital episodes with a procedure | 636,924 | 626,988 | 9,936 | 93,943 | 90,703 | 3,240 | 542,981 | 536,285 | 6,696 |
| Hospital episodes per 1000 | 101.0 | 100.7 | 130.2 | 14.9 | 14.6 | 42.5 | 86.1 | 86.1 | 87.7 |
| Procedures | 1,221,149 | 1,201,845 | 19,304 | 166,368 | 160,271 | 6,097 | 1,054,781 | 1,041,574 | 13,207 |
| Procedures per 1,000 people | 193.7 | 193.0 | 252.9 | 26.4 | 25.7 | 79.9 | 167.3 | 167.2 | 173.0 |
| Procedures per person with a procedure | 2.5 | 2.5 | 2.6 | 2.0 | 2.0 | 2.3 | 2.5 | 2.5 | 2.5 |
| Procedures per hospital episode | 1.9 | 1.9 | 1.9 | 1.8 | 1.8 | 1.9 | 1.9 | 1.9 | 2.0 |

Table S2a Top ten procedure blocks within each procedure block group, comparing mental health service users (MH) to other NSW residents (No MH): Cardiovascular, Gastrointestinal and Respiratory procedure blocks. Adjusted incidence rate ratio (aIRR) after standardisation for age, sex, and socioeconomic disadvantage. Block not shown and/or standardised rates not calculated when total procedures in MH group < 20.


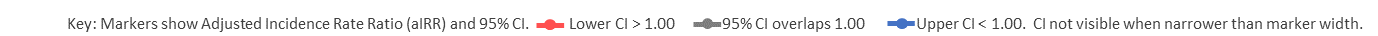


Notes: (MH) Mental Health. (aIRR) Adjusted Incidence Rate Ratio. (CI) Confidence Interval. (ACHI) Australian Classification of Health Interventions. (CABG) Coronary Artery Bypass Graft. (LIA) Left Internal Mamillary Artery. (VV) Varicose Veins (Ins) Insertion. (Perm) Permanent. (W) with. (R/O) removal of. (FB) Foreign body.

Table S2b Top ten procedure blocks within each procedure group, comparing mental health service users (MH) to other NSW residents (No MH): Muskuloskeletal, Skin and Plastics, Genito-urinary procedure blocks. Adjusted incidence rate ratio (aIRR) after standardisation for age, sex, and socioeconomic disadvantage. Block not shown and/or standardised rates not calculated when total procedures in MH group < 20.


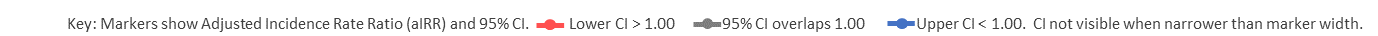


Notes: (MH) Mental Health. (aIRR) Adjusted Incidence Rate Ratio. (CI) Confidence Interval. (ACHI) Australian Classification of Health Interventions. (R/O) removal of. (w) with. (M/S) Musculoskeletal. (Agt) Agent. (Proc) Procedure. (SSCT) skin and subcutaneous connective tissue. (Manip) Manipulation. (Ext) Extraction. (Ins) Insertion.

Table S2c Top ten procedure blocks within each procedure group, comparing mental health service users (MH) to other NSW residents (No MH): Nervous system, Eye, Nose and Mouth procedure blocks. Adjusted incidence rate ratio (aIRR) after standardisation for age, sex, and socioeconomic disadvantage. Block not shown and/or standardised rates not calculated when total procedures in MH group < 20.


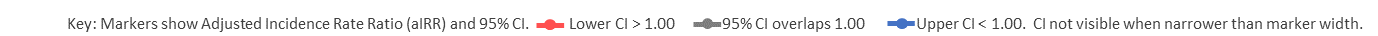


Notes: (MH) Mental Health. (aIRR) Adjusted Incidence Rate Ratio. (CI) Confidence Interval. (ACHI) Australian Classification of Health Interventions. (R/O) removal of. (Ins) Insertion. (Proc) Procedure. (W) with. (Perc) Percutaneous. (Str) Structures. (Vert) Vertebral. (I/V) Intervertebral. (Agt) Agent. (Elect) Electrode.

Table S2d Top ten procedure blocks within each procedure group, comparing mental health service users (MH) to other NSW residents (No MH): Gynaecological, Breast, Endocrine, Blood-forming organ procedure blocks. Adjusted incidence rate ratio (aIRR) after standardisation for age, sex, and socioeconomic disadvantage. Block not shown and/or standardised rates not calculated when total procedures in MH group < 20.


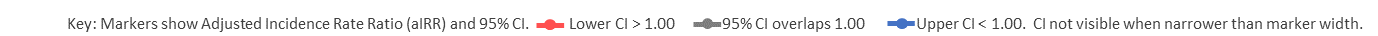


Notes: (MH) Mental Health. (aIRR) Adjusted Incidence Rate Ratio. (CI) Confidence Interval. (ACHI) Australian Classification of Health Interventions. (Rep) Repair. (Pelv flr) Pelvic floor.

S3: Subgroup analysis, surgical procedure rates and counts in people with Severe and Persistent Mental Illness (SPMI), other mental health service users (Other MH service MH) and the rest of the NSW adult population (No MH service use). Crude rates and adjusted rates after standardisation by age, sex and socioeconomic disadvantage.

|  |  |  | Rate (per 1000 person years) | |
| --- | --- | --- | --- | --- |
| Group | Procedures | Person-years | Crude | Standardised (95% CI) |
| All procedures |  |  |  |  |
| No MH service use | 1,201,845 | 6,228,641 | 193.0 | 192.7 (192.3 - 193.0) |
| SPMI | 6,311 | 34,410 | 183.4 | 213.5 (207.7 - 219.2) |
| Other MH service | 12,993 | 41,910 | 310.0 | 373.0 (366.2 - 379.8) |
| Planned procedures |  |  |  |  |
| No MH service use | 1,041,574 | 6,228,641 | 167.2 | 167.0 (166.6 - 167.3) |
| SPMI | 4,548 | 34,410 | 132.2 | 157.6 (152.6 - 162.6) |
| Other MH service | 8,659 | 41,910 | 206.6 | 247.1 (241.6 - 252.7) |
| Emergency procedures | |  |  |  |
| No MH service use | 160,271 | 6,228,641 | 25.7 | 25.7 (25.6 - 25.8) |
| SPMI | 1,763 | 34,410 | 51.2 | 56.2 (53.3 - 59.1) |
| Other MH service | 4,334 | 41,910 | 103.4 | 125.9 (121.9 - 129.8) |

S4: Subgroup analysis, separately comparing people with Severe and Persistent Mental Illness and Other Mental Health Service Users (MH) to the rest of the NSW adult population (No MH), showing incidence rate ratios (aIRR) adjusted for age, sex and socioeconomic disadvantage.

Notes: (MH) Mental Health subgroup. (No MH) other NSW residents. (aIRR) Adjusted Incidence Rate Ratio. (CI) Confidence Interval. aIRR not calculated where number of procedures in MH subgroup is less than 20.
